# Over-Expression of VvWRKY1 in Grapevines Induces Expression of Jasmonic Acid Pathway-Related Genes and Confers Higher Tolerance to the Downy Mildew

**DOI:** 10.1371/journal.pone.0054185

**Published:** 2013-01-14

**Authors:** Chloé Marchive, Céline Léon, Christian Kappel, Pierre Coutos-Thévenot, Marie-France Corio-Costet, Serge Delrot, Virginie Lauvergeat

**Affiliations:** 1 Univ. Bordeaux, ISVV, EGFV, UMR 1287, F-33140 Villenave d’Ornon, France; 2 INRA, ISVV, EGFV, UMR 1287, Villenave d’Ornon, France; 3 Univ. Poitiers, UMR CNRS 7267, Ecologie et Biologie des Interactions, équipe Physiologie Moléculaire du Transport de Sucres, Bat. B31, Poitiers, France; 4 INRA, UMR SAVE-1065, ISVV, BP 81, Villenave d’Ornon, France; Friedrich-Alexander-University Erlangen-Nurenberg, Germany

## Abstract

Most WRKY transcription factors activate expression of defence genes in a salicylic acid- and/or jasmonic acid-dependent signalling pathway. We previously identified a *WRKY* gene, *VvWRKY1*, which is able to enhance tolerance to fungal pathogens when it is overexpressed in tobacco. The present work analyzes the effects of *VvWRKY1* overexpression in grapevine. Microarray analysis showed that genes encoding defence-related proteins were up-regulated in the leaves of transgenic *35S::VvWRKY1* grapevines. Quantitative RT-PCR analysis confirmed that three genes putatively involved in jasmonic acid signalling pathway were overexpressed in the transgenic grapes. The ability of VvWRKY1 to *trans*-activate the promoters of these genes was demonstrated by transient expression in grape protoplasts. The resistance to the causal agent of downy mildew, *Plasmopara viticola*, was enhanced in the transgenic plants. These results show that VvWRKY1 can increase resistance of grapevine against the downy mildew through transcriptional reprogramming leading to activation of the jasmonic acid signalling pathway.

## Introduction

One of the most damaging pathogens for grapevine is the causal agent of downy mildew, *Plasmopara viticola*, an obligate biotrophic oomycete that infects all green parts of the plant. To maintain the yield and the quality of grapes and their processed products (wine, juice and dried fruit), vine growers usually spray costly phytochemicals. The environmental impact of these chemicals together with the development of fungicide resistant strains requires the development of alternative methods for disease control. To this end, it is useful to characterize the molecular mechanisms leading to the activation of grapevine defence responses.

The plant immune system is divided into two interconnected branches depending on the pathogen perception mechanism [1]. The first one is the PTI (PAMP-triggered immunity) where transmembrane receptors activate a basal defence mechanism upon recognition of conserved pathogen-associated molecular patterns (PAMPs). The second one is defined as the ETI (effector-triggered immunity) in which pathogen effectors are detected inside the cells by NB-LRR resistance proteins encoded by most *R* genes. ETI often results in induction of hypersensitive (HR) cell death thought to limit the access of the pathogen to water and nutrients.

After recognition of the invading pathogen, PTI induction of phosphorylation cascades through MAP kinase signalling [2] activates transcription factors controlling genes which encode proteins involved in defence processes, such as pathogenesis-related (PR) proteins and enzymes of secondary metabolism. This pathway is tightly tuned by phytohormones including salicylic acid (SA), jasmonic acid (JA) and ethylene (ET), but also abscisic acid (ABA) and gibberellins [3]. These molecules are involved in signalling pathways that interact extensively. For example, SA and JA are antagonists for the activation of many genes and are involved in different mechanisms of defence. Plant resistance to biotrophic pathogens is classically believed to be mediated through SA signalling and often induces hypersensitive response (HR) followed by Systemic Acquired Resistance (SAR). Upon attack by a necrotrophic pathogen, insect, or after wounding, JA accumulates and induces a different set of defence responses such as the accumulation of secondary metabolites (alkaloids, phenolic compounds, terpenes) and of PR proteins.

The PTI and ETI responses require large-scale transcriptional reprogramming coordinated by transcription factors belonging to different families [4]. Among them, WRKY proteins represent one of the largest superfamily of transcription factors in higher plants, after the R2–R3 MYB family [5]. Most characterizations of WRKY proteins demonstrated the critical role of these transcription factors as activators or repressors of plant innate immunity [6]. Nonetheless, emerging functions of some WRKY proteins in other processes (germination, senescence, development and abiotic stress) are also considered. The majority of Arabidopsis *WRKY* genes are up-regulated upon pathogen infection or treatment with defence elicitors and signalling molecules [4], [6]. The effects observed on disease resistance in overexpressor and silenced lines suggest that tight regulation of *WRKY* transcript accumulation is required for efficient defence against various pathogens, with regard to the specificity of a given *WRKY* gene, a given pathogen and putative redundancy within the WRKY family.

Most WRKY transcription factors studied have been shown to be involved in SA and/or JA signalling pathways. Constitutive expression of *AtWRKY33* conferred increased resistance to necrotrophic pathogens but caused enhanced susceptibility to the bacterial pathogen *Pseudomonas syringae*. This phenotype is associated with opposite expression of JA- and SA-regulated genes, suggesting that AtWRKY33 is a key component of the crosstalk between signalling pathways involved in responses to pathogens developing different mechanisms of pathogenesis [7], [8]. Interestingly, a pair of allelic genes *OsWRKY45-1* and *OsWRKY45-2*, play opposite roles in rice-bacteria interactions through different activations of signalling pathways. OsWRKY45-1 modulates SA and JA levels whereas OsWRKY45-2 only significantly induces JA levels [9]. The first steps of WRKY proteins activation after elicitation involve MAP kinase pathways [10], [11], but only few additional components of the transduction cascade have been identified.

We previously identified two grapevine genes, *VvWRKY1* [12] and *VvWRKY2* [13], encoding WRKY transcription factors belonging to the groups II and I respectively [6]. *VvWRKY1* is up-regulated in leaves in response to various treatments, such as ergosterol [14], SA, ethephon, and H_2_O_2_ [12], suggesting this transcription factor plays a role in these defence-related signalling pathways. A preliminary functional characterization of VvWRKY1 was performed by overexpression in tobacco, but the molecular mechanism leading to a better tolerance of the *35S::VvWRKY1* transgenic plants to *Pythium*, powdery mildew and downy mildew were not elucidated in this heterologous system. Physical interaction and functional regulation by VvWRKY1 of a *VvLTP* promoter, a grapevine PR14 involved in JA signaling pathway, were demonstrated previously conducting to a better tolerance against *Botrytis* [14], [15]. The present study investigates the role of VvWRKY1 in the homologous context, by generating *VvWRKY1* overexpressing grapevines. Transcriptomic analyses mainly highlighted defence-related genes and photosynthesis-related genes, providing clues on VvWRKY1 involvement in plant defence. Furthermore, some of the up-regulated genes in the transgenic plants encode proteins putatively involved in JA signalling. Transient transformation of grapevine protoplasts showed that VvWRKY1 activates their promoters. To investigate the role of VvWRKY1 against pathogens, transformed and WT grapevines were challenged with *Plasmopara viticola*, the causal agent of the downy mildew.

## Results

### Generation of Transgenic Grapevines Overexpressing VvWRKY1

**Figure 1:**
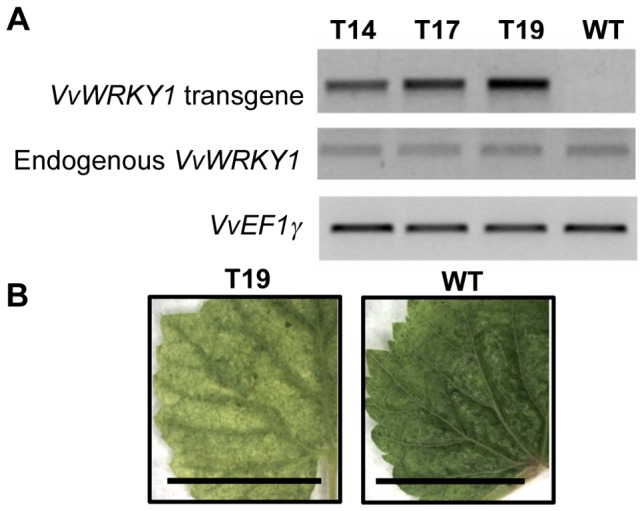
Figure **1.** **Characterization of transgenic grapevine plantlets overexpressing** ***VvWRKY1*** **.** (A) Transcript levels of *VvWRKY1* transgene and endogenous *VvWRKY1* gene in three transgenic lines and the WT line. Total RNA were extracted from leaves of *in vitro* grown plants and semi-quantitative RT-PCR were performed. *VvEF1γ* was used as a reference gene. (B) Leaves from the T19 transgenic line and regenerated 41B (WT) *in vitro* grown plants. Scale bars correspond to 1 cm.

To determine whether VvWRKY1 regulates defence responses in the homologous species, transgenic grapevine plants that constitutively overexpress *VvWRKY1* were generated. Ten kanamycin-resistant plantlets regenerated from grapevine 41B cell suspension cultures were obtained. The presence of the transgene was confirmed by PCR reactions carried out on genomic DNA extracted from leaves using a forward primer specific to *VvWRKY1* and the reverse primer designed in the *NOS* terminator (data not shown). The expression level of *VvWRKY1* was then evaluated by semi-quantitative RT-PCR. Three lines exhibiting different levels of transgene expression were chosen for further studies (Figure 1A). PCR run with primers located in the 3′UTR of VvWRKY1 (not present in the integrated transgene), did not detect any change in the expression of endogenous *VvWRKY1* expression in these lines compared to control ones.

Moreover, growth and development of the transgenic lines did not differ significantly from that of WT control plantlets, except that leaf colour was slightly paler (Figure 1B and Figure S1).

### Transcriptional Profiling of the Transgenic Grapevines Reveals that VvWRKY1 is Involved in Several Defence Mechanisms

In order to identify the genes transcriptionally regulated by VvWRKY1, which encompass direct and indirect targets of this transcription factor, microarray hybridizations comparing the transcriptomes of T19 and of control plants were carried out. Analysis of two biological replicates and their corresponding dye swap, revealed that 160 genes were differentially expressed in transgenic grapevines (two-fold change threshold and non-adjusted *P*-value <0.01). This low number of genes is probably due to the stringency of the analysis and the fact that the two replicates were conducted totally independently. Only the genes showing a significant difference of transcript accumulation in both independent experiments were kept for further analysis.

Among those 160 genes, 101 and 59 were respectively down- and up-regulated in the T19 line compared to 41B control leaves. However, 14 probes showed no match or multiple matches with V1 version of the Pinot Noir grapevine genome (Table S1). Functional annotation was then performed by mapping the probes to the *V. vinifera* Gene Index or the Pinot noir grapevine genome [16] and by BLAST comparison to plant sequences. A significant homology with known genes could be found for 92 of the differentially expressed genes (61 down-regulated and 31 up-regulated). Functional categorization of those genes using MapMan classification indicates that 18 categories were represented (Figure S2 and Table S1). Nine categories are represented in overexpressed genes, whereas under-expression concerns 17 categories.

This classification highlighted a strong over-representation of photosynthesis-related genes and a smaller but significant one for genes encoding proteins involved in the regulation of transcription and protein synthesis/modification. Interestingly, all the differentially expressed genes putatively associated with primary metabolism were down-regulated in the transgenic plants. Among them, we found genes putatively encoding different components of the two photosystem reaction centers, several chlorophyll a/b binding proteins, light harvesting complex proteins, ATP synthase delta chain, chloroplast ferredoxin-NADP^+^ oxydoreductase, oxygen evolving enhancer protein 1, a carbonic anhydrase, a vacuolar invertase and a pyruvate kinase-like protein (Table S1).

Among the 21 genes classified into transcription and protein synthesis/modification, around 50% were either over- or under-expressed (Figure S2). Three genes encoding transcription factors potentially related to stress responses were overexpressed: an AP2/ERF domain containing protein, a GRAS family transcription factor and a WRKY transcription factor (Table 1; Table S1).

**Table 1:** The twenty most up-regulated genes in *35::VvWRKY1* grapevine leaves (line T19) compared to wild-type.

Probe ID	Putative function	Fold induction	*P*-value
Vv_10011539	JAZ1/TIFY10A (JASMONATE-ZIM-DOMAIN protein1) protein binding (JAZ1.1)	4.74	3,00E-05
Vv_10011339^a^	JAZ1/TIFY10A (JASMONATE-ZIM-DOMAIN protein1) protein binding (JAZ1.2)	3.80	8,00E-05
Vv_10009335	SNF1-related protein kinase regulatory beta subunit 1	3.30	0.00015
Vv_10010692**^b^**	Calcium-binding protein	3.09	0.00013
Vv_10012824	Putative nitrate transporter (NRT3.1)	3.09	0.00013
Vv_10010045	Unknown function, homologous to At3g54000	2.97	0.00238
Vv_10011290	Xyloglucan endotransglucosylase/hydrolase	2.92	0.00629
Vv_10014155	Protein serine/threonine phosphatase (PP2C)	2.89	0.00326
Vv_10000951	Lipoxygenase (LOXO)	2.82	0.00019
Vv_10009788	Putative cinnamyl alcohol dehydrogenase (CAD)	2.81	0.00042
Vv_10007607	ATP binding/ATPase1 protein	2.79	0.00021
Vv_10008748	AP2/ERF domain containing TF/Dehydration-responsive element binding protein 3	2.77	0.0002
Vv_10010712	Xyloglucan endotransglucosylase/hydrolase	2.75	0.00125
Vv_10011253	Caffeic acid O-methyltransferase (COMT)	2.74	0.00387
Vv_10008629	GRAS family transcription factor/Chitin-inducible gibberellin-responsive protein 1	2.69	0.00024
Vv_10002324	Gibberellin 2 oxydase	2.67	0.00024
Vv_10011044**^b^**	Calcium-binding protein	2.63	0.00045
Vv_10013131	ATSEC20	2.64	0.0044
Vv_10010418	Beta-1,3-glucanase (PR2)	2.58	0.00029
Vv_10000253^a^	JAZ1/TIFY10A protein binding	2.57	0.00028

Sequences showing significant homology with known genes are only presented here. The grapevine genome identifier (G12X ID and CRIBI annotation) associated with each sequence is given in Table S3. Letters in superscript indicate probes that hybridize the same gene.

Eight out of the 11 cell-wall related genes encode cell-wall modifying enzymes, such as pectinesterase, xylosidase and pectin methylesterase inhibitor. All of them are down-regulated with the exception of three genes encoding xyloglucan endotransglucosylase/hydrolases (XTH; Table 1).

Using this categorization, most of the genes up-regulated in the *35S::VvWRKY1* T19 line can be gathered in stress-related categories including cell wall modification (three XTH, see above), secondary metabolism, hormone metabolism, signalling, and stress-regulated (Figure S2 and Table 1).

Two genes related to phenylpropanoid metabolism were up-regulated in the transformed grapevine: a *cinnamyl alcohol deshydrogenase* and a *caffeic acid O-methyl transferase* gene, which encode proteins controlling key steps in the lignin biosynthesis pathway (Table 1). Among the stress-related genes, it is interesting to notice that a *β-1,3 glucanase* gene was up-regulated. A thaumatin-like encoding gene was also detected (Table S1). The protein encoded shows similarity to VvTL1, a PR-5 class protein [17].

Among the twenty most overexpressed genes (Table 1), the presence of genes encoding a calcium-binding protein, a SNF1-related protein kinase regulatory β-subunit and a protein serine/threonine phosphatase (PP2C) can be underlined. These three genes can be related to stress-responsive signalling pathways.

Seven genes related to hormone metabolism were differentially expressed in *35S::VvWRKY1* plants. Among them, two genes coding for gibberellin 2-oxidases involved in GA catabolism are regulated in an opposite way. Interestingly, the other five genes up-regulated in *VvWRKY1* overexpressors are related to JA metabolism and signalling. They include two *lipoxygenase* genes (*LOXO* and *LOXA*) involved in JA biosynthesis [18] and two genes encoding proteins highly similar to Arabidopsis JAZ1/TIFY10A and JAZ2/TIFY10B (between 58 to 61% similarity) (one of the genes being represented by two probes in the microarray chip), which are transcriptional repressors of JA signalling [19]. These genes correspond to the two most up-regulated genes in the *VvWRKY1* overexpressing line (Table 1).

### VvWRKY1 Transactivates Promoters of Genes Related to Jasmonate Signalling

**Figure 2:**
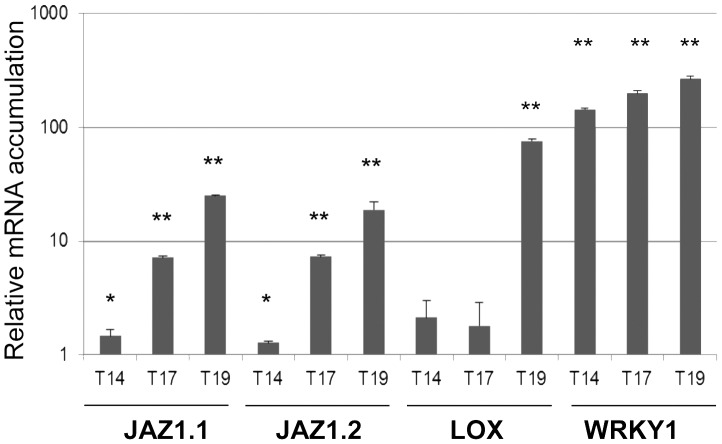
Quantitative RT-PCR analysis of *VvJAZ1.1*, *VvJAZ1.2, LOX* and *VvWRKY1* transcripts accumulation in transgenic grapevine plants. mRNA accumulation was assessed by quantitative RT-PCR in leaves of three plants for each line. Results were expressed as means and standard deviation relative to control plant, the expression of which has been assigned the value  = 1 on the logarithmic scale (Student’s *t* test; * *P*<0.05, ** *P*<0.01 versus WT).

The microarray analysis suggested that *VvWRKY1* overexpression in grapevine affects several genes involved in jasmonate signalling pathway. To validate these data, the two *JAZ1/TIFY10A* genes named *JAZ1.1* for VIT_09s0002g00890 and *JAZ1.2* for VIT_11s0016g00710 were selected together with the *13-lipoxygenase* (*LOXO*) gene, and their transcript levels were investigated in the three transgenic lines previously chosen (T14, T17 and T19) using RT-PCR. The transcript levels of both *JAZ1.1* and *JAZ1.2* were significantly higher in transgenic grapevine leaves of the three independent lines than in wild-type plants (Figure 2). The level of induction of these two genes varied among the transgenic lines and could be correlated to *VvWRKY1* transgene expression. The *LOXO* gene was significantly overexpressed only in line T19. A slight induction could also be seen in the two other transgenic lines, but it appeared not significant compared to WT.

**Figure 3:**
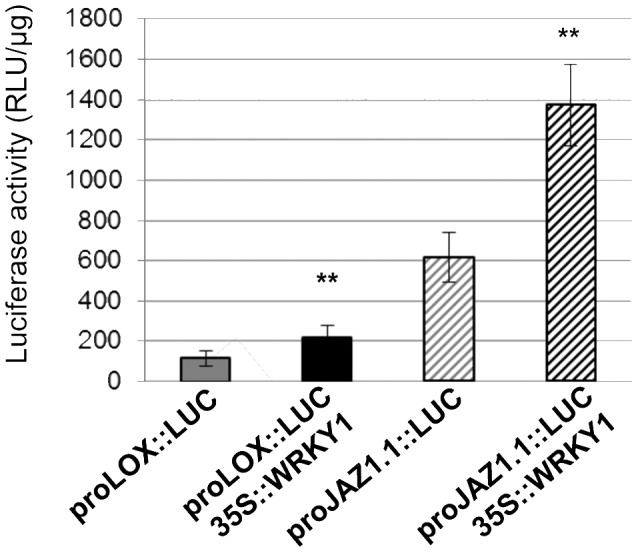
VvWRKY1 activates the promoter of *VvLOX* and *VvJAZ1.1* genes in grapevine protoplasts. Transient transformation of grapevine protoplasts was achieved using the pLuc reporter vector under control of either the *LOX* promoter or the *JAZ1.1* promoter alone, or each of these constructs was co-transfected with the *35S::WRKY1* as the effector vector. As negative control, protoplasts were co-transformed with the pLuc reporter vector without promoter and the *35S::WRKY1* effector plasmid and showed no luciferase activity. Each column represents the mean ± standard deviation of four independent transfection experiments (Student’s *t* test; ** *P*<0.01 *versus* activity measured in protoplasts transformed with the corresponding reporter construct alone).

To further investigate the possible regulation of these genes by VvWRKY1, approximately 1000 bp of the corresponding promoter sequences were isolated from Cabernet Sauvignon genomic DNA. Unfortunately, the corresponding sequence of *JAZ1.2* promoter could not be amplified, may be due to differences between Pinot noir and Cabernet Sauvignon sequences. *JAZ1.1* and *LOX* promoters have been sequenced and possess seven and four W-boxes (TGAC core), respectively. Trans-activation assays of these promoters by VvWRKY1 have been performed in Cabernet Sauvignon grapevine protoplasts. Both promoters were significantly trans-activated by about two-fold when protoplasts were co-transformed with *35S::VvWRKY1* (Figure 3). These results demonstrate that VvWRKY1 can transiently activate these promoters in grapevine cells.

### VvWRKY1 Overexpression Confers a Lower Susceptibility to Grapevine Downy Mildew

**Figure 4:**
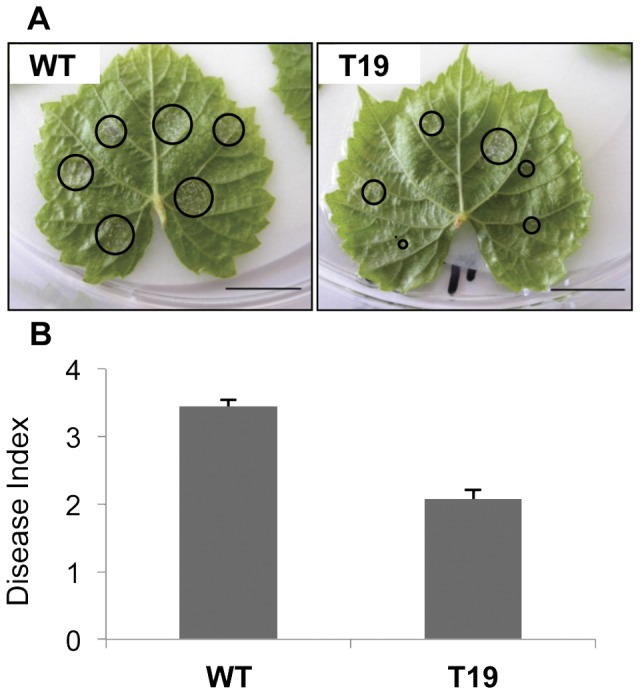
Downy mildew infection of the transgenic grapevines. Leaves of WT and transgenic T19 *in vitro*-grown plants were detached and infected on the abaxial leaf surface by a spore suspension. The intensity of sporulation was evaluated 7 days after inoculation. (A) Representative images of WT and T19 infected leaves. Circles highlight the surface covered by the downy mildew sporulation at each inoculation site, seven days after infection. Bars correspond to 1 cm. (B) *Plasmopara viticola* development was evaluated visually using a disease index from 0 to 5. The average of each infection site notation is represented. Bars correspond to the standard error and the asterisk indicates a significant difference from wild-type (Student’s *t* test, *P*<0.001).

To investigate whether *VvWRKY1* overexpression confers resistance/tolerance to pathogens, the T19 and the control lines were challenged with *P. viticola*. Since the asexual reproductive cycle occurs within six to seven days, the degree of infection was evaluated using a disease index seven days later. Two independent experiments gave very similar results. Figure 4 details the results of one of those experiments. The number of infection sites on which the oomycete grew and sporulated was smaller in the T19 transgenic line than in the WT (Figure 4A). The disease evaluation gave a disease index of 3.44 and 2.07 for the WT and the T19 lines respectively (Figure 4B), also revealing a lower susceptibility of the *VvWRKY1* overexpressor compared to the WT. This significant difference corresponds to a 40% decrease of susceptibility in the T19 line. Furthermore, the number of sporangia evaluated after resuspension into water showed an average of 1400 and 908 spores per inoculation site for the WT and T19 lines respectively, thus confirming the previous visual evaluation of oomycete progression. It corresponds to a 36% decrease of sporulation on the transgenic line compared to the WT, which is very close to the previous 40% decrease of the surface covered by mycelium. However the difference observed by spores counting was not statistically significant, mainly due to the fact that several leaves were pooled for the measurement, thus lowering the replicate number. Taken together, these data indicate that overexpression of *VvWRKY1* in the T19 line confers a lower susceptibility to *P. viticola.*

## Discussion

Improvement of grapevine resistance against phytopathogens is a major economic and environmental issue. Several genes/genomic regions playing an important role in this resistance have been identified [20], [21], [22]. We previously demonstrated that *VvWRKY1* improves resistance to pathogenic fungi when it is overexpressed in tobacco [12]. The present work shows that overexpression of *VvWRKY1* in its homologous system, grapevine, induces a global transcriptional reprogramming which clearly enhanced resistance to downy mildew. Transcript levels were compared between a *35S::VvWRKY1* transgenic line and control plants using one of the *V. vinifera* microarray available at the beginning of this work, covering 50% of grapevine coverage (14562 probes). Even if the transcriptional changes do not necessarily reflect changes in protein, the global transcriptome analysis allowed us to identify the direct and indirect targets of VvWRKY1 transcription factor in a constitutive expression context.

First, this genomic analysis shows a decrease of transcript levels of genes related to primary metabolism and particularly 21 photosynthesis-associated genes. A decrease of photosynthesis activity is often observed in plants grown *in vitro*, due to exogenous supply of sucrose which does not necessitate the normal development of photosynthetic apparatus [23]. However, photosynthesis-related gene expression is lower in transgenic plants than in control ones. Solexa sequencing highlighted a significant down-regulation of transcripts associated with photosynthesis in *Vitis amurensis* grapevine leaves infected with *P. viticola* [24]. In some cases, a similar decrease is correlated with the level of resistance to *P. viticola* [25], [26]. Thus, the decrease in photosynthesis-associated genes expression observed here might be a secondary effect of defence-related transcriptional reprogramming induced by *VvWRKY1* overexpression.

Secondly, most of the up-regulated genes in 35S::VvWRKY1 can be related to defence mechanisms. In particular, transcriptomic analysis, validated by qRT-PCR as well as promoter transactivation showed that VvWRKY1 was involved in JA-signalling regulation. The oxygenation of α-linoleic acid by 13-lipoxygenases (13-LOX) is the initial step in JA formation by the LOX pathway [27]. *LOX* transcripts rapidly accumulate in response to wounding and pathogen challenge, and subsequently results in increased levels of jasmonates. The two *13-LOX* genes which are up-regulated in *35S::VvWRKY1* plants correspond to *LOXO* and *LOXA*, previously identified in *V. vinifera* Sauvignon Blanc berries [18]. The strong expression of *LOXO,* in seeds, correlates with a high concentration of JA in that tissue. However, in berries subjected to wounding or infection by *Botrytis cinerea*, *LOXO* expression was induced whereas *LOXA* transcripts accumulation was decreased.

Our transcriptomic analysis also showed that overexpression of VvWRKY1 activates the transcription of two *JAZ* genes, *VvJAZ1.1* and *VvJAZ1.2.* At low JA levels, JAZ proteins suppress the activity of transcription factors involved in the regulation of early JA-responsive genes. In the presence of active form of JA, JAZ proteins are ubiquitinated by the SCF^COI1^ complex, and subsequently degraded by the 26S proteasome. This activates these transcription factors, allowing expression of early response genes including JA-responsive transcription factors, and the *JAZ* genes themselves [28]. The negative feedback loop created by elevating gene expression of repressors then attenuates the JA signal [19]. *VvJAZ1.1* and *VvJAZ1.2* were also identified in *V. rupestris* [29] and named *VrJAZ2/TIFY10b* and *VrJAZ1/TIFY10a,* respectively. Interestingly, *JAZ1.2* gene is strongly up-regulated in resistant *V. amurensis* after infection with *P. viticola* [24]. *JAZ* genes have been recently identified as WRKY targets in Arabidopsis [8], [30]. In the Arabidopsis *wrky18 wrky40* double mutants, five *JAZ* genes are up-regulated compared to WT in uninfected plants [30]. The *in vivo* binding of WRKY40 to the *JAZ8* promoter was confirmed using chromatin immunoprecipitation assays (ChIP). ChIP qPCR experiments also suggested a direct negative regulation of *JAZ1* and *JAZ5* by WRKY33 upon infection [8].

Interestingly, the promoter regions of *LOXO* and *JAZ1.1* genes contain four and seven W-boxes respectively. This W-boxes enrichment is significantly higher than the statistical expectation of a random distribution of the motif, as described by [31] who found an average of 4.3 W-boxes per 1.1 Kb promoter sequence of the *PR1* regulon promoters, and it suggests a transcriptional regulation involving WRKY transcription factors. Promoter activation assays in grapevine protoplasts confirmed the ability of VvWRKY1 to *trans*-activate both promoters.

WRKY transcription factors are known to regulate the expression of Pathogenesis Related (PR) genes through a SA- or JA/ET-dependent pathway. Transcript levels of *VvPR2*, encoding a β-1,3 glucanase, and *VvPR5*, encoding a thaumatin-like protein (VvTL1), are increased in *35S::VvWRKY1* plants compared to WT. The induction of the rice thaumatin-like Rtlp1 by SA, JA and an elicitor from the rice blast fungus depends on the presence of W-boxes within its promoter [32]. Interestingly, compared to WT plants, mutants in the closest Arabidopsis *VvWRKY1* homologue, *AtWRKY75*, exhibited reduced and delayed induction of *PR1*, *PR2* and *PR5* in response to *Pseudomonas syringae* infection [33]. Moreover, a study conducted on a grapevine segregating population issued from a Merzling×Teroldego cross showed a significant transcriptional activation of *PR2* and *PR5* genes in *P. viticola* resistant genotypes [34].

Therefore, our results suggest that upon elicitation/infection, *VvWRKY1* transcripts accumulate and the VvWRKY1 transcription factor can bind, among others, *LOX* gene promoters and activate their transcription. These transcriptional responses lead to induce JA biosynthesis and subsequently JA-responsive gene expression. Together with other transcriptional responses, this JA pathway will eventually participate in establishing grapevine downy mildew resistance. Moreover, we may speculate that the concomitant JAZ transcript accumulation by VvWRKY1 might act for a negative feedback loop regulation.

Interestingly, the involvement of jasmonic acid in grapevine resistance to downy mildew is also supported by previous studies. Particularly, the work that compared transcriptional responses to downy mildew infection in a susceptible (*V. vinifera*) and a resistant (*V. riparia*) grapevine species highlighted a strong up-regulation of several *WRKY* genes as well as JA biosynthesis-related genes (13-LOX, omega-3 fatty acid desaturase, allene oxide cyclase, allene oxide synthase) in the resistant genotype which was correlated with an increase in JA and MeJA contents [25]. In accordance to our results, several other signal transduction pathways including calcium signalling, ethylene signalling, MAP kinases, phosphatases, receptor-like proteins and numerous transcription factors were also affected. Additionally, priming of defence responses by β-amino butyric acid (BABA) or sulfated laminarin (PS3), which increase resistance against *P. viticola*, was strongly reduced by the LOX inhibitor (ETYA), suggesting that both PS3 and BABA effects are mediated through the JA pathway [35], [36].

In summary, we showed that overexpression of *VvWRKY1* in grapevine results in up-regulation of several genes associated with the activation of defence-related signalling events (calcium signalling, phosphorylation/dephosphorylation), monolignol biosynthesis and *PR* gene expression. Moreover, when it is stably or transiently overexpressed in grapevine, VvWRKY1 activates the expression of genes related to JA synthesis and responses, such as *LOX* gene expression, leading eventually to higher level of downy mildew tolerance. Further studies will be needed to identify the set of direct targets of VvWRKY1.Our results provide the first evidence for the involvement of a grapevine WRKY transcription factor, VvWRKY1, in tolerance to downy mildew, one of the most devastating grapevine diseases. As the 41B genotype is used as rootstock, it should be important to confirm that *VvWRKY1* overexpression can also improve resistance in a *Vitis vinifera* scion genotype. Nonetheless, these results suggest some clues to improve fungal pathogen resistance in grapevine.

## Materials and Methods

### Grape Transformation from Embryogenic Cell Suspension Cultures

*VvWRKY1* cDNA sequence (GenBank accession number: AY585679) was isolated from a grape berry cDNA library (*Vitis vinifera* L. cv. Cabernet Sauvignon) at the veraison stage [12]. The pGiBin19 *35S::VvWRKY1* binary vector, containing the *VvWRKY1* coding sequence, was described previously [12]. This plasmid was introduced into the EHA 105 *Agrobacterium tumefaciens* strain which was used to transform grape cells. Embryogenic cells derived from the 41B rootstock (*Vitis vinifera* cv Chasselas × *Vitis Berlandieri*) were used for grapevine stable transformation. They were maintained and transformed according to the protocol described in [37]. WT control plants are 41B plantlets that have been regenerated from embryogenic suspension cells in the same conditions as transgenic plants.

The grape cell suspension used for transient expression assay was derived from *Vitis vinifera* Cabernet Sauvignon berries. It was maintained in the dark at 25°C on an orbital shaker (100 rpm) and weekly subcultured in a medium described by [38].

### Gene Expression Analysis by Semi-quantitative PCR and Real-time Quantitative PCR

Total RNA was isolated from leaves of four *in vitro* grown plants for each line by LiCl precipitation method [12]. The first-strand cDNA was generated from two µg of RQ1 DNase-treated RNA using MMLV Reverse Transcriptase (Promega) with oligo(dT)_18_ primers, according to the manufacturer’s protocol. Semi-quantitative RT-PCR amplifications were performed as described in [12]. The experiments were performed at least twice with similar results. *VvWRKY1* transgene mRNA was amplified using a 5′ specific primer and a 3′primer designed in the *NOS* terminator sequence. *VvWRKY1* endogenous mRNA was amplified using the same 5′specific primer and a 3′primer designed in a part of the 3′UTR not present in the transgene.

To validate microarray expression profiles, quantitative real-time PCR reactions were performed using SYBR Green on an *iCycler iQ®* (Bio-Rad), according to the procedure described by the supplier. Reactions were performed in triplicate using 0.2 µM each primer, 5 µL SYBR Green mix (Bio-Rad) and 0.8 µL DNAse-treated cDNA in a final volume of 10 µL. Data were normalized according to the *VvEF1*γ (AF176496) gene expression levels using the algorithms outlined by [39]. Primer sequences and size of amplified products are listed in Table S2.

### Microarray Experiments

#### Microarray hybridization and data acquisition

Microarrays were produced using the *Vitis vinifera* Array-Ready Oligo Set™ Version 1.0 (Operon Biotechnologies) as described in [40]. Two biological replicates were used to perform the RNA profiling of the transgenic *35S::VvWRKY1* line 19 grapevine (T19) compared to the wild type plantlets. For each one, one microarray hybridization and its dye swap were done. The data are available in ArrayExpress (http://www.ebi.ac.uk/arrayExpress) under the accession number E-MTAB-1077.

For each replicate and for each line analyzed (T19 and WT), all leaves from at least three *in vitro* grapevine plantlets were harvested. Total RNA was prepared and DNase-treated as described above. Twenty micrograms of RNA from WT and T19 lines were reverse-transcribed, labeled with amino-allyl dUTP, purified and fluorescently labeled with CyDye NHS ester molecules (Cy3 and Cy5) using CyScribe Post-labelling Kit (Amersham) according to the instructions except some slight modifications. The cDNA were obtained and the aminoallyl dUTP were incorporated using two consecutive reactions of reverse transcription by the “CyScript reverse transcriptase”. A purification step was performed with the CyScribe GFX purification kit (Amersham). Each cDNA sample was then coupled with a different fluorophore Cy3 or Cy5. Incorporation of fluorescent dyes and cDNA concentration were evaluated using the spectrophotometer Ultrospec 3100 Pro (Amersham). The hybridization conditions are described in [41] except that hybridization was performed at 42°C for 16 h.

Microarray data analysis was performed according to [40]. Genes with expression ratio above 2 and *P*-value below 0.01 were considered as differentially expressed.

#### Probe mapping and gene annotation

Oligonucleotides probes were mapped to the initial 12X version of the grapevine genome [16] and the V1 version of the CRIBI available at http://ddlab.sci.univr.it/FunctionalGenomics/. Genome sequences were annotated using best blast matches against the uniref100 database using qualifiers described in Table S3. Annotations for differentially expressed genes were checked manually. Gene associations to the MapMan Ontology [42] were verified manually for differentially expressed genes.

#### Promoter transactivation assay in grapevine protoplasts

The putative promoter sequences of *VvLOX* (VIT_09s0002g01080) and *VvJAZ1.1* (VIT_09s0002g00890) genes were identified in the grapevine genome sequence (http://www.genoscope.cns.fr/externe/GenomeBrowser/Vitis/) and primers were designed to amplify an approximate 1000 bp DNA fragment. PCR amplification was performed on *V. vinifera* L. cv. Cabernet Sauvignon genomic DNA using Phusion™ High-Fidelity DNA Polymerase (Ozyme). Restriction sites were introduced into the primers (Table S1) to allow the cloning of these promoters into the pLuc plasmid [43]. Those sequences were deposited in GenBank (accession numbers: JN226403 and JN226404). To obtain the effector vector, the β-glucuronidase (*GUS*) gene of the pGreen-35S::GUS (http://www.pgreen.ac.uk/) was replaced by the cDNA sequence of *VvWRKY1* between *Sal*I and *Not*I restriction sites.

Isolation and PEG-mediated transfection of grapevine protoplasts were performed as described in [44]. Ten µg of each plasmid was used for transformation. After transformation, the protoplasts were pelleted and homogenized in 80 *µ*L of 1X Passive Lysis Buffer (PLB, Promega), by grinding and centrifuged. Protein content was determined in the supernatant using the Bradford assay. Ten µL was used to measure Firefly luciferase activity using the dual-luciferase reporter assay kit (DLR, Promega) by addition of 50 µL LARII reagent. Light emission was measured with a Luminova 1254 luminometer (BIO ORBIT) for 10 sec with an initial delay of 2 sec. Luciferase activities were reported as relative light unit (RLU) per µg of proteins. All transfection experiments were performed in triplicate and each set of promoter experiments was repeated with similar relative ratios to the respective control. The experiment was repeated three times.

### Plant Infection with *Plasmopara viticola* (Downy Mildew)

*Plasmopara viticola* (strain COU 15, INRA, Bordeaux, France) was maintained on the abaxial surface of grapevine (*Vitis vinifera* L. cv. Cabernet Sauvignon) leaf disks at -20°C and sub-cultured twice for seven days before the assay. Sporangia were collected with a paint brush and suspended in sterile water. The concentration was adjusted to 15×10^3^ spores per mL.

Detached grapevine leaves from six-week-old *in vitro* plants were placed upside down in Petri dishes on a wet and sterile filter paper. Inoculation was performed by depositing four to twelve 15 µL-droplets onto the abaxial face of each leaf surface, depending of the leaf size. The experiment was done twice independently. For the experiment shown, 39 WT-leaves and 38 T19-transgenic leaves were treated that allowed the inoculation at 261 and 257 sites, respectively. The inoculated leaves were incubated in Petri dishes under controlled conditions (22°C), 80% humidity, with a 16 h light day. Seven days after, inoculation disease intensity was estimated by measuring growth and intensity of mycelium and sporulation, as described previously [45]. In a second time, sporangia were resuspended by vortexing four to five infected leaves in three mL of water, and spores were then counted using Malassez cells under a microscope.

## Supporting Information

Figure S1 **Pictures of** ***in vitro*** **grown plantlets.** A) Untransformed 41B line, B) Transgenic 35S::VvWRKY1 line.
(DOCX)

Figure S2 **Functional categorization of genes differentially expressed in** ***35S::VvWRKY1*** **plants compared to wild type plants (** ***P*** **-value 0.05 and threshold 2).** The 96 genes showing a good homology with known genes were only considered. BIN categories are indicated on the abscissa. Gene associations to the MapMan Ontology were verified manually. Down-regulated genes are represented by white bars and up-regulated genes by grey bars.
(DOCX)

Table S1 **Genes with a two-fold differential expression in** ***35S::VvWRKY1*** **plants.** One hundred sixty probe sets showed more than two-fold differential expression between *VvWRKY1* overexpression line and control plants. Grapevine genome identifier (G12X ID) and CRIBI identifier (CRIBI ID) are indicated.
(XLSX)

Table S2 **Sequences of primers used in this study.** F, forward; R, reverse. Genbank accession or CRIBI Genome Browser numbers are indicated.
(DOCX)

Table S3 **Sequence annotation qualifiers.**
(DOCX)
