# Supplementary material for: Over-Expression of VvWRKY1 in Grapevines Induces Expression of Jasmonic Acid Pathway-Related Genes and Confers Higher Tolerance to the Downy Mildew
Source: PLoS One. 2013 Jan 14;8(1):e54185. doi: 10.1371/journal.pone.0054185 (PMC3544825; doi:10.1371/journal.pone.0054185)

**Figure S1: Pictures of *in vitro* grown plantlets.** A) Untransformed 41B line, B) Transgenic 35S::VvWRKY1 line.

A

B


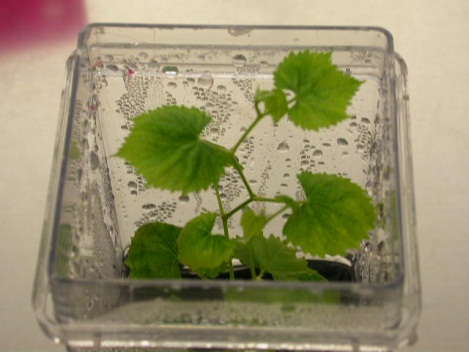

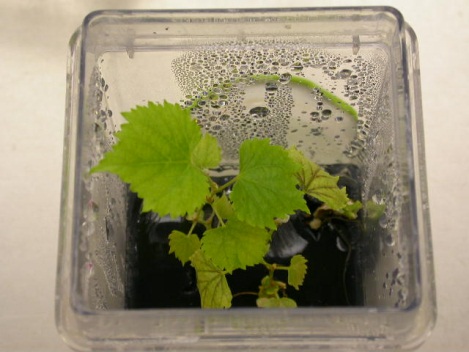

Supplement: Figure S1 — Pictures of in vitro grown plantlets. A) Untransformed 41B line, B) Transgenic 35S::VvWRKY1 line. (DOCX) [file pone.0054185.s001.docx]
