# Supplementary material for: Over-Expression of VvWRKY1 in Grapevines Induces Expression of Jasmonic Acid Pathway-Related Genes and Confers Higher Tolerance to the Downy Mildew
Source: PLoS One. 2013 Jan 14;8(1):e54185. doi: 10.1371/journal.pone.0054185 (PMC3544825; doi:10.1371/journal.pone.0054185)
Supplement: Figure S2 — Functional categorization of genes differentially expressed in 35S::VvWRKY1 plants compared to wild type plants ( P -value 0.05 and threshold 2). The 96 genes showing a good homology with known genes were only considered. BIN categories are indicated on the abscissa. Gene associations to the MapMan Ontology were verified manually. Down-regulated genes are represented by white bars and up-regulated genes by grey bars. (DOCX) [file pone.0054185.s002.docx]

**Figure S2: Functional categorization of genes differentially expressed in *35S::VvWRKY1* plants compared to wild type plants** (*P*-value 0.05 and threshold 2). The 96 genes showing a good homology with known genes were only considered. BIN categories are indicated on the abscissa. Gene associations to the MapMan Ontology were verified manually. Down-regulated genes are represented by white bars and up-regulated genes by grey bars.

Number of genes
