# Supplementary material for: Over-Expression of VvWRKY1 in Grapevines Induces Expression of Jasmonic Acid Pathway-Related Genes and Confers Higher Tolerance to the Downy Mildew
Source: PLoS One. 2013 Jan 14;8(1):e54185. doi: 10.1371/journal.pone.0054185 (PMC3544825; doi:10.1371/journal.pone.0054185)
Supplement: Table S2 — Sequences of primers used in this study. F, forward; R, reverse. Genbank accession or CRIBI Genome Browser numbers are indicated. (DOCX) [file pone.0054185.s004.docx]

**Table S2: Sequences of primers used in this study.** F, forward; R, reverse. Genbank accession or CRIBI Genome Browser numbers are indicated.

| **Names and accession numbers** | | **Primers (5’-3’)** | **Size of amplified product (bases)** |
| --- | --- | --- | --- |
| ***Semi-quantitative and quantitative RT-PCR*** | | | |
| *VvWRKY1 transgene* | | F: GAGAATGATATGGAAAGAGTGG  R: TCATCGCAAGACCGGCAACA (3’NOS) | 235 |
| *VvWRKY1 gene* (AY585679 ; VIT_17s0000g01280) | | F: GAGAATGATATGGAAAGAGTGG  R: CATTCGTTCTCAGACACAATA | 319 |
| *VvEF1γ *(AF176496 ; VIT_12s0035g01130) Semi-Q PCR | | F : GCGGGCAAGAGATACCTCAA  R : TCAATCTGTCTAGGAAAGGAAG | 257 |
| *VvEF1γ *(AF176496 ; VIT_12s0035g01130) | | F: CAAGAGAAACCATCCCTAGCTG  R: TCAATCTGTCTAGGAAAGGAAG | 91 |
| *VvJAZ1.1* (VIT_09s0002g00890) | | F: Cctccacaggttcttggag  R: ggtttgtgaggacctgcagg | 91 |
| *VvJAZ1.2* (VIT_11s0016g00710) | | F : ttcaccggttcttggagaag  R : gtgtcctcttcaggctttgg | 100 |
| ***Cloning (restriction sites are indicated in bold)*** | | | |
| *LOXO Promoter* (VIT_09s0002g01080) | F : AAT**GGATCC**TCTTTTGAGATTCAACTTTATCC  R : aat**tctaga**CTGTGATCTTTACAAGAAATAG | | 1007 |
| *JAZ1.1 Promoter* (VIT_09s0002g00890) | F : AAT**GGATCC**GAAGTTGGAGTTGGAGTTGG  R: AAT**CCATGG**TTCTTCACGGCGACTAGTTAA | | 953 |
