# Supplementary material for: Over-Expression of VvWRKY1 in Grapevines Induces Expression of Jasmonic Acid Pathway-Related Genes and Confers Higher Tolerance to the Downy Mildew
Source: PLoS One. 2013 Jan 14;8(1):e54185. doi: 10.1371/journal.pone.0054185 (PMC3544825; doi:10.1371/journal.pone.0054185)
Supplement: Table S3 — Sequence annotation qualifiers. (DOCX) [file pone.0054185.s005.docx]

**Table S3: Sequence annotation qualifiers.**

| **Criterion** | **Qualifier** |
| --- | --- |
| > 50% alignment identity | homologue to |
| > 70% alignment identity | similar to |
| ≤ 70% alignment identity | weakly similar to |
| > 98% hit coverage | complete |
| ≤ 98% hit coverage | partial |
